# *Translocase of Outer Mitochondrial Membrane 40*, as a Promising Biomarker for the Diagnosis of Polycystic Ovary Syndrome and Pan-Cancer

**DOI:** 10.1007/s43032-024-01652-2

**Published:** 2024-07-26

**Authors:** Xin Zhang, Lin Zhu, ChenHao Ma, Shu-Ning Zhao, Chu-Yue Zhao, Hui Sun

**Affiliations:** 1 Department of Laboratory Diagnosis, The Second Affiliated Hospital of Harbin Medical University, Harbin, 150086 China; 2 Department of Obstetrics and Gynecology, Center for Reproductive Medicine, Guangdong Provincial Key Laboratory of Major Obstetric Diseases, Guangdong Provincial Clinical Research Center for Obstetrics and Gynecology, Guangdong-Hong Kong-Macao Greater Bay Area Higher Education Joint Laboratory of Maternal- Fetal Medicine, The Third Affiliated Hospital of Guangzhou Medical University, Guangzhou, China; 3 Key Laboratory for Reproductive Medicine of Guangdong Province, The Third Affiliated Hospital of Guangzhou Medical University, Guangzhou, China; 4 Pharmaceutical Experiment Teaching Center, College of Pharmacy, Harbin Medical University, NO.194, BaoJian Street, Nan Gang District, Harbin, Heilongjiang 150081 P. R. China

**Keywords:** PCOS, Pan-cancer, *TOMM40*, Machine learning

## Abstract

Polycystic ovary syndrome (PCOS) is a metabolic disease that affects the reproductive system, and its pathogenesis remains unresolved. Through the application of bioinformatics and molecular biology techniques, this study has identified a significant association between translocase of outer mitochondrial membrane 40 (*TOMM40*) and both PCOS and pan-cancers. The selection of PCOS biomarkers included *TOMM40*, which we found to be significantly decreased in the PCOS group both in vitro and in vivo, using molecular biology methods such as Western Blot as well as immunohistochemistry. Over-expression *TOMM40* can rescue the effect on apoptosis rate and proliferation suppression induced by DHEA in KGN cells. *TOMM40* as a biomarker for the diagnosis of PCOS. The pan-cancer analysis revealed an association between elevated *TOMM40* expression in Uterine Corpus Endometrial Carcinoma and an unfavorable prognosis, while increased *TOMM40* expression in six tumor types was linked to a favorable prognosis. Therefore, *TOMM40* can be regarded as a promising biomarker for diagnosing both PCOS and pan-cancer.

## Introduction

PCOS is a metabolic disorder affecting the reproductive system [1]. In PCOS, immature and degenerating follicles accumulate, ovarian tissue expands excessively, androgen levels rise, and insulin levels rise [2, 3]. Furthermore, PCOS can be triggered by insulin resistance, disruptions of metabolism, and imbalances of LH (Luteinizing hormone) and FSH (Follicle-stimulating hormone) [4]. PCOS and gynecologic cancers often share common risk factors, such as obesity and hormonal imbalances. The mRNA and microRNA patterns of individuals with PCOS exhibited striking resemblances to various types of cancers, including ovarian cancer [5], breast cancer [6], pancreatic cancer [7], endometrial cancer and other forms of cancer [8]. These pieces of evidence highlight the potential risk of increased incidence of gynecological cancers associated with PCOS, and the molecular mechanisms may play a role in both cancer and PCOS patients.

In recent times, the connection between PCOS and the advancement of cancer has been a widely discussed subject in research studies due to the association with metabolic abnormalities, which generate an elevated susceptibility to developing cancers [9, 10]. It is known that PCOS is a significant risk factor for endometrial cancer and may increase the risk of ovarian cancer in younger women. [11] Cancers and PCOS are complex illnesses caused by a combination of genetic, internal, hormonal imbalances, metabolic disruptions, and environmental influences [12]. Hence, there is an urgent need to enhance our comprehension of the physio pathologic mechanism that governs these intricate molecular impacts. This will facilitate the advancement of new medications and enhance the prognosis of these individuals.

*TOMM40* coding for the gene translocase of outer mitochondrial membrane 40, is an essential component of the mitochondrial *TOMM40* complex responsible for facilitating the import of proteins into mitochondria [13]. Numerous research studies have indicated that the *TOMM40* gene might play a role in increasing the likelihood of developing cancer [14]. Such as, the discovery of *TOMM40* from Rui Sun et al. provides a fresh perspective on the development and outlook of endometrial cancer [15]. According to Alandejani SA et al., Cheng Y et al [16] stated that *TOMM40* serves as a marker for mitochondrial dysfunction in Alzheimer’s disease, while also highlighting the differential transcription of TOMM40 in the brain. Nevertheless, the precise way *TOMM40* genetic mutations elevate the susceptibility to PCOS, and various types of cancer remains undisclosed.

Through the use of public databases such as the Cancer Genome Atlas (TCGA) and Gene Expression Omnibus (GEO) and the LASSO and SVM-RFE models, we employed to study the molecular mechanisms of existing PCOS treatments, we can gain a deeper understanding of the disease. This knowledge helps prioritize targets for more effective therapies. Such as Surleen Kaur [17] discovered that the expression of specific genes in PCOS tissues was associated with metabolic disorders and oxidative stress, suggesting there may be an association between PCOS and cancer. Bioinformatics software has been used in cancer research to examine and identify metabolism-related genes. According to the author Yang and colleagues [18], people with ovarian cancer may be able to treat their disease with *CCNB2*, *TYMS*, *KIF11*, *KIF4A*, *BUB1B*, *FOXM1*, and *CDC20*. However, it is still uncertain if these central genes are exclusively implicated in the progression of PCOS.

## Methods and Materials

### Gathering and Analyzing Data

Using TCGA database, *TOMM40* expression is determined in various tissues. In order to obtain normal human tissue data, we downloaded the Genotype-Tissue Expression (GTEx) database from the UCSC Xena database at https://xenabrowser.net/datapages/. As a result, we obtained the matrix of relative expression data that was divided by the total number of reads, and the data in log2 (TPM + 1) format were then analyzed. The abbreviations section contained all the lists of cancer abbreviations. In cancers, the changes in the genetic makeup, positioning, and communication of *TOMM40* are observed. An analysis of the HPA database (http://www.proteinatlas.org) was conducted to determine how much *TOMM40* is expressed in human tumors. In order to demonstrate *TOMM40* PPIs, the String database (https://string-db.org/) and the ComPPI link group (http://comppi.linkgroup.hu/) were used.

### Analysis of Prognostic and Functional Enrichment

There were three factors that predicted outcomes: overall survival (OS), progression-free survival (PFS), and disease-specific survival (DSS). In our study, we used Kaplan-Meier and Cox regression to assess the association between *TOMM40* and pan-cancer. As part of the evaluation, the Normalized Enrichment Fractions (NES) and False Discovery Rates (FDR) of each cancer were calculated using JavaScript. ClusterProfiler and GSVA have been used for the gene enrichment analysis, and ggplot has been used to visualize the results as heat maps.

### Immune Infiltration of ***TOMM40***

Several factors contribute to tumor initiation and progression, including the tumor microenvironment (TME). Through the analysis of immune score, stromal score, and ESTIMATE Score, it was observed that an increase in the Immune Score or Stromal Score corresponded to a higher proportion of the immune matrix, indicating a positive correlation with immune infiltration. It is calculated by adding the Immune Score and Stromal Score together, which indicate the overall proportional component of the ESTIMATE Score’s duration. Using the R package ‘ggplot2’, we analyzed the correlation between *TOMM40* mRNA levels and various immune cell subsets in different types of cancer. There were a few subsets of cells included in these studies: Fibroblasts, B cells, neutrophils, endothelial cells (Endo), g/d T cells, monocytic lineage, myeloid dendritic cells, Cytotoxic lymphocytes, CD8^+^ T cells, Neutrophils as well as NK cells. The results were visualized in a heatmap.

### Gene Ontology Analysis

Biological process (BP), cellular components (CC), and molecular function (MF) characteristics of genes, gene products, and sequences were extensively characterized using gene ontology (GO) analysis. A GO analysis was conducted on DEGs (differential gene expression) using Metascape (http://metascape.org/gp/index.html), selecting gene sets with a nominal *P* value below 0.05 as enriched.

### Clinical Specimens

The Reproductive Medicine Center at The Third Affiliated Hospital of Guangzhou Medical University collected follicular fluid from patients who had undergone in vitro fertilization (IVF) or intracytoplasmic sperm injection (ICSI). The oocyte was collected 36 h after the human chorionic gonadotropin (HCG) triggered ovulation in the mature follicles. All samples were collected with written informed consent. The Rotterdam criteria were used to diagnose 10 cases of PCOS. The normal group (consisting of 10 cases) included patients with infertility caused by issues related to the fallopian tubes or male factors.

### Animals

Female mice of the C57BL/6 J strain, aged 6 weeks, and weighing between 15 and 20 g, were acquired from Changsheng Animal Technology Co Ltd., located in Shenyang, China. In the experiment, two groups of animals were used: mices in the sham group were subcutaneously injected with 1.5 mg/g sesame oil. Mices in the DHEA group were injected with 1.5 mg/kg of DHEA (diluted in 0.1 ml sesame oil). After 21 days, the mice were anesthetized and sacrificed by cervical dislocation [19, 20]. The modeling technique was explained earlier [21, 22]. Studies on animals were approved by the Institutional Animal Care and Use Committee at Harbin Medical University, Harbin, China (Cat. IRB3016722).

### The Isolation, Extraction, and Cultivation of GCs

After oocyte retrieval, follicular fluid was collected after oocyte extraction under the microscope. The follicular fluid was rotated in a centrifuge, after centrifugation, the deposited upper layer cells were extracted and then mixed with an equal amount of DMEM/F12 culture medium by previous study [23]. GCs were obtained by centrifuging the cell suspension and then transferring it to a solution containing 50% Percoll (GE) at a volume ratio of 2:3. An erythrocyte lysate is used to remove erythrocytes. After the cells are removed, they may be used for cultivating, isolating proteins, or extracting RNA, or they can be stored at a temperature of -80 °C. Cultured cells were suspended in DMEM/F12 (Gibco, 11,330,057) medium supplemented with 10% FBS (Cas9x, FBP-X0220) in 12-well plates. An incubator at 37 °C with 5% CO_2_ concentration was then used to incubate the plate. The medium was then replaced after 24 h with another one.

### Extraction of Proteins and Performing Immunoblotting

Proteins were extracted from granulosa cells (GCs) using RIPA lysate, followed by denaturation in loading buffer at 95 °C for 5 min. Subsequently, the extracted proteins underwent immunoprotein blotting according to standardized protocols. Primary antibodies specific to the target proteins were employed: *TOMM40* (Cat. A3213, Abclonal, China), *Bcl-2* (Cat. 381,702, Zenbio, China), ki67 (Cat. A15595, Abclonal, China), PCNA (Cat. 350,190, Zenbio, China), *Cleaved caspase 3* (Cat. A2516, Abclonal, China), *BAX* (Cat. R22708, Zenbio, China), and *β-actin* (Cat. GB15003, Servicebio, China). Secondary antibodies from Servicebio, China, were utilized for detection.

### AO/EB Staining

The AO/EB double fluorescent staining kit (Cat. R20292, Yuanye Biotechnology, China) was utilized for apoptosis detection. Initially, cells were collected and washed with PBS, then resuspended in an appropriate volume of PBS. The cell concentration was adjusted to 0.2-5 × 10^6 cells/ml. Subsequently, the cell suspension was mixed with the staining working solution at a 25:1 ratio and incubated at room temperature for 5–15 min. Apoptotic cells were quantified in five randomly selected fields of view using a microscope (Phenix, ShangRao, China).

### Colony Formation

Cells were seeded at a density of 1 × 10^3^ cells per well in 6-well dishes and cultured at 37 °C for 14 days. Following incubation, cells underwent three washes with PBS, each lasting 15 min. Subsequently, the cells were fixed with 4% formaldehyde and stained with 0.1% crystal violet for 30 min. Colony counting was performed by selecting five random fields for each sample.

### Extraction of RNA Followed by RT-PCR

Trizol was used to extract total RNA from GCs, and reverse transcription was achieved using a TaKaRa kit according to the manufacturer’s instructions. A ^△^CT value was determined by amplifying reverse transcribed cDNA. Two [A-B]-[C-D] (A: average CT value of target genes in the experimental group, B: average CT value of reference genes in the experimental group, C: Average CT value of target genes in the control group, D: average CT value of reference genes in the control group) were used to calculate and compare the results. Here are the serial numbers for the primers as follows:

TOMM40-Forward- CCTCCAGAGCATCACGCCTTG,

TOMM40-Reverse- TGACAGTGCCCTCCTCTCCAG.

GAPDH-Forward-5’- CAGGAGGCATTGCTGATGAT-3’,

GAPDH-Reverse-5’- GAAGGCTGGGGCTCATTT-3’.

### Cell Growth

KGN cells, a GC line, were obtained from the Riken Cell Bank (Wako, Saitama, Japan). Based on the experimental design, the wells were categorized into four groups: Control group, DHEA group, DHEA + Empty vector group, and DHEA + *TOMM40* group. Each group had five replicate wells. The dishes were placed in an incubator at a temperature of 37 °C with 5% CO_2_ for a duration specified by the experimental plan. Prior to each test, a serum-free solution with 10% CCK-8 (cell counting kit-8) was prepared, and 100mL of the prepared solution was added to each well. The entire procedure was conducted in a light-proof environment. The dishes were placed in a light-proof environment and left to incubate for a duration of 2 h. The final measured value was obtained by subtracting the measured OD value from the blank group, and the statistical software was used to plot the cell proliferation curve.

### Flow Cytometry

Cells were digested with trypsin without EDTA and centrifuged at 300 g for 5 min at 2–8 °C. Post-centrifugation, cells were washed twice with precooled PBS and resuspended in 400 µL of Annexin V binding buffer. Subsequently, 5 µL of Annexin V-FITC staining solution was added to the cell suspension, followed by incubation at 2–8 °C for 15 min in the dark. For propidium iodide (PI) staining, 5–10 µL of PI solution was mixed gently into the cell suspension and incubated for an additional 5 min at 2–8 °C in the dark. Finally, the samples were analyzed using flow cytometry immediately after staining.

### ELISA Kit

Serum levels of sex hormones, including estradiol (E2), progesterone (P), and follicle-stimulating hormone (FSH), were quantified using sandwich ELISA kits (R&D Systems, USA). The ELISA kits were prepared, and assays conducted according to the manufacturer’s instructions [24, 25].

### Statistical Analysis

The data was analyzed using SPSS software version 26.0. The clinical data that followed a normal distribution was analyzed using an independent test, while the clinical data that did not follow a normal distribution was analyzed using a non-parametric test (with the 25th and 75th percentiles representing the indicators that did not conform to the normal distribution). The statistical significance was observed when the *P* < 0.05.

## Results

### Using Machine Learning to Detect the Possible Indicators of PCOS

**Fig. 1:**
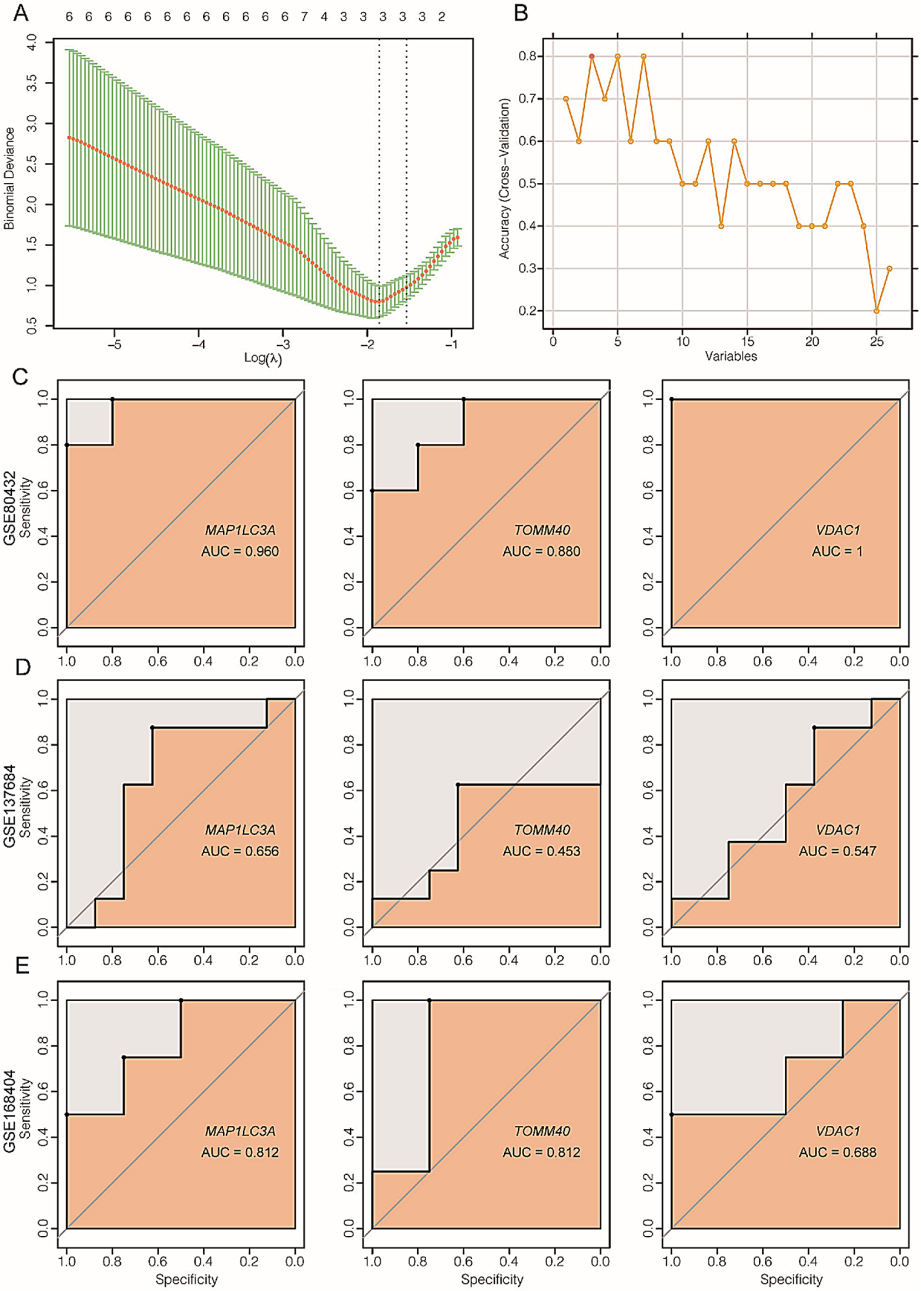
Machine-learning algorithm used to screen for potential biomarkers of PCOS. **(A)** Screening for biomarkers using Lasso regression. The optimal number of genes (*n* = 6) corresponds to the lowest point on the curve. **(B)** Screening for biomarkers using the SVM-RFE algorithm. **(C-E)** The ROC area of *MAP1LC3A*, *TOMM40* and *VDAC1* in GSE80432, GSE137684 and GSE168404

We identified three shared genes, namely *MAP1LC3A*, *TOMM40*, and *VDAC1*, using two machine learning algorithms: the LASSO model and the SVM-RFE model. Afterward, the AUC value of the ROC curve was utilized to assess the classification performance of the three genes in the independent datasets GSE80432 and GSE137684.Combined with the training dataset GSE168404, the average AUC value of *MAP1LC3A* in the three datasets is 0.809, while the average AUC values of *VDAC1* and *TOMM40* were 0.715 and 0.745, respectively. According to our understanding, *MAP1LC3A* has demonstrated a significant contribution to the advancement of PCOS.(Fig. 1)

### Expression of ***TOMM40*** in PCOS

**Fig. 2:**
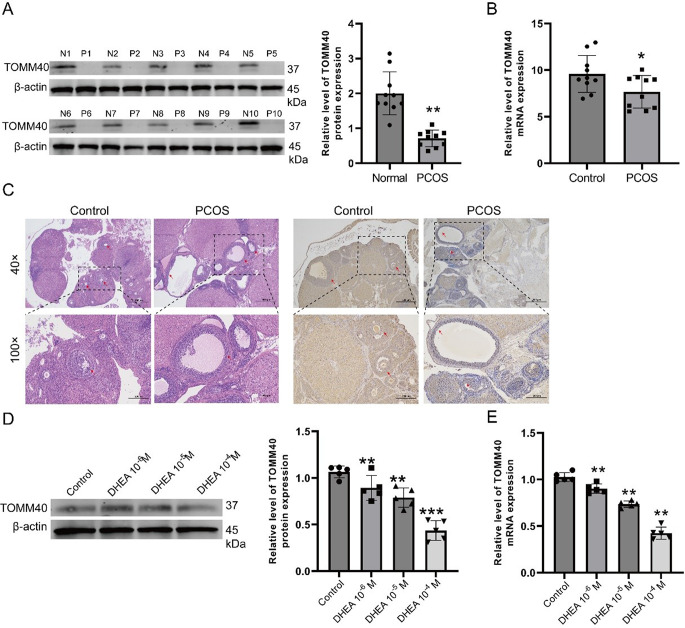
In PCOS, the level of *TOMM40* expression is depicted. **(A-B)** Detection of *TOMM40* expression in the granulosa cells of patients with PCOS was performed using western blotting and qRT-PCR. **(C)** H&E staining and immunohistochemistry was used to assess the presence of *TOMM40* in ovarian tissues of individuals with PCOS and the control group in Rat. **(D-E)** Western blotting and qRT-PCR were used to detect the levels of *TOMM40* expression in KGN cells following treatment with DHEA in a dose-dependent manner. Significance was observed at* *P* < 0.05, ** *P* < 0.01 or ***P < 0.001, when compared to the control group

To explore the biological functions of *TOMM40* in PCOS. Initially, we examined the *TOMM40* expression in GEO datasets (GSE80432, GSE137684, and GSE168404). In GSE168404, the level of *TOMM40* expression was found to be reduced in PCOS (*P* < 0.05, Fig. S1A). Nevertheless, there was no noticeable variation in *TOMM40* in the GSE80432 and GSE137684 datasets, possibly because of the limited number of samples (Fig. 2B, C and Fig. S1 B-C). Next, we obtain granulosa cells from the ovaries of 10 patients with PCOS and healthy volunteers for the purpose of examining the expression of *TOMM40* through qRT-PCR and western blotting. In PCOS, the data indicated a decrease in *TOMM40* expression (Fig. 2A-B). Furthermore, we first established PCOS model (*n* = 5, Fig. S2 A-C) to test the expression of *TOMM40* by immunohistochemistry. Our data shown the amount of *TOMM40* was reduced in mice ovarian tissue with PCOS (Fig. 2C). Subsequently, we used DHEA treatment KGN cells as a vitro model for PCOS to show the role and expression of *TOMM40* [20, 26, 27]. We follow the application of DHEA at different concentrations (0, 10^− 6^,10^− 5^ or 10^− 4^ M) for a duration of 48 h, the levels of *TOMM40* mRNA and protein were assessed. The findings indicated that the concentration-dependent effect of DHEA led to a reduction in the expression of *TOMM40*, as observed in Fig. 2D-E, in comparison to the DHEA 0 M group.

### The Excessive Expression of *TOMM40* Repressed the DHEA-induced Inhibition of KGN Cell Proliferation

**Fig. 3:**
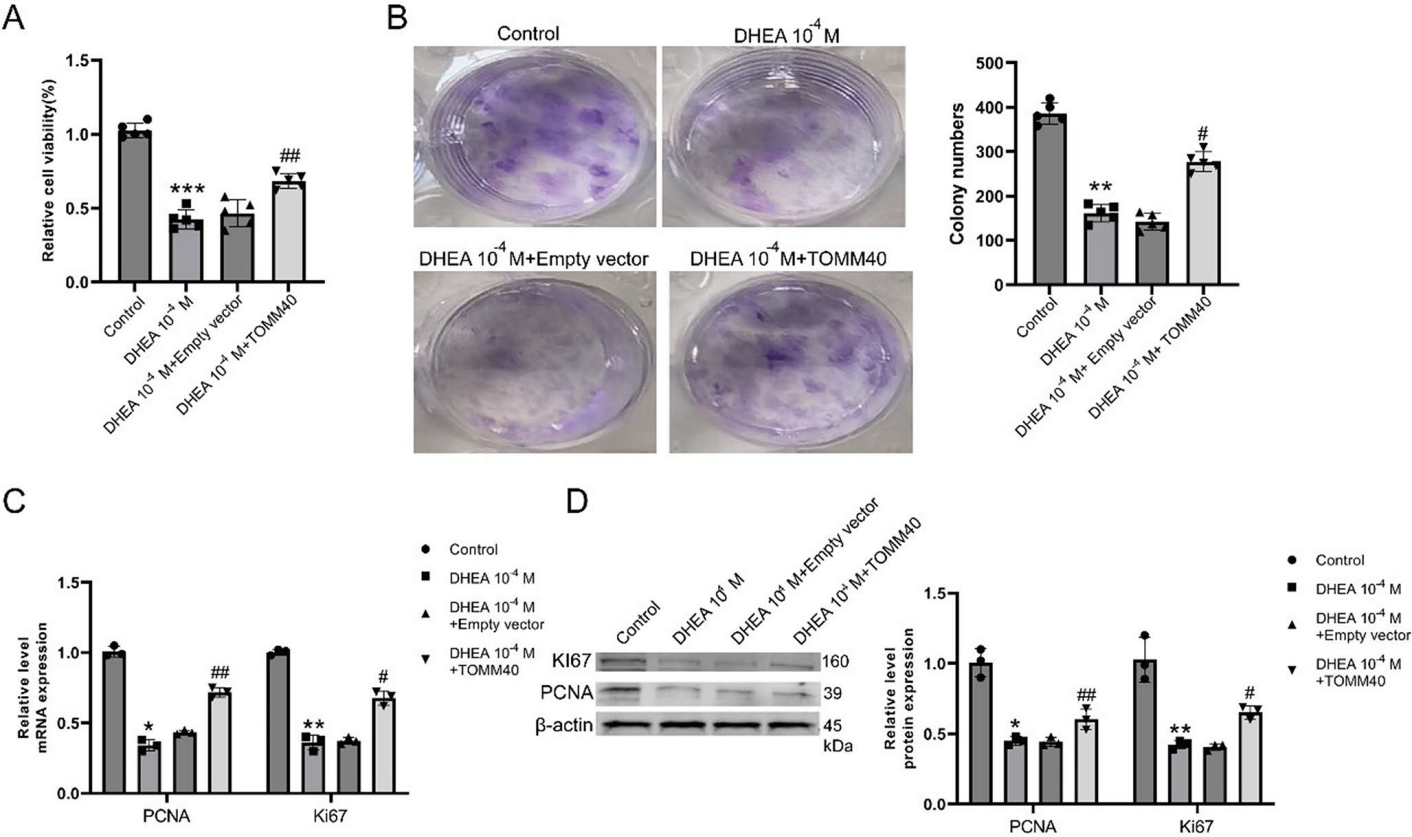
*TOMM40* administration promoted the proliferation of DHEA-induced ovarian granulosa cells. **(A)** Using CCK-8, cells were identified as proliferating. **(B)** Colony-building. **(C-D)** qRT-PCR and western blotting were used to determine PCNA and KI67 levels. Significant difference (***P < 0.001) compared to the Control group. Significance was observed at ^#^*P* < 0.05, ^##^*P* < 0.01, and ^###^*P* < 0.001 when compared to the DHEA 10^− 4^ M group

Compared to the control (DHEA 0 M), the cell viability to proliferation was reduced in the DHEA 10^− 4^ M group, but this was partially reversed by the over-expression of *TOMM40* (Fig. 3A). According to colony-forming analysis, it is evident that the over-expression of *TOMM40* can enhance colony formation, a process that is hindered by DHEA as shown in Fig. 3B. Furthermore, the reduced levels of *PCNA* and *Ki67* in KGN cells induced by DHEA 10^− 4^ M were subsequently increased following *TOMM40* intervention (Fig. 3C-D). Clearly, *TOMM40* affects the growth of DHEA-stimulated KGN cells.

**Fig. 4:**
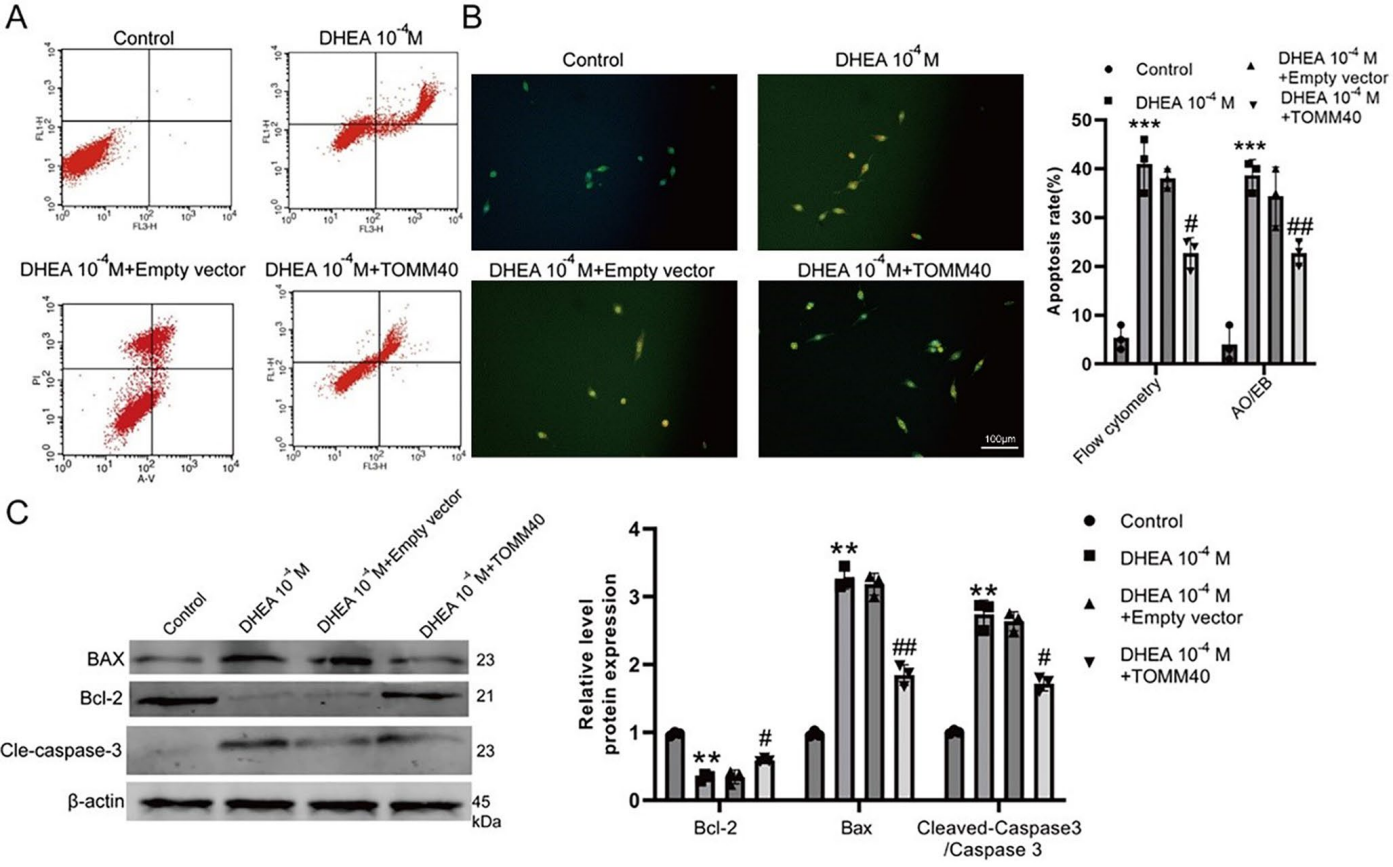
*TOMM40* administration inhibited the apoptosis of DHEA-induced ovarian granulosa cells. **(A-B)** The rate of programmed cell death was measured through flow cytometry analysis and AO/EB staining. **(C)** Protein levels of Bcl‐2, Bax, and Cleaved-caspase3 were assessed through western blot analysis. *** *P* < 0.001, ** *P* < 0.01versus control group. # *P* < 0.05, ##*P* < 0.01 versus DHEA or DHEA-empty vector group

Flow cytometry analysis was used to assess cell apoptosis, while the evaluation of related proteins was conducted through AO/EB staining and western blotting. In contrast to DHEA 0 M, the presence of DHEA 10^− 4^ M significantly heightened the apoptosis rate of KGN cells. Conversely, the introduction of *TOMM40* over-expression had the opposite effect, as evidenced by the decreased apoptosis in the presence of DHEA 10^− 4^ M + TOMM40-OE (Fig. 4A-B). Moreover, the decreased Bcl-2 levels and increased levels of Bax and cleaved-caspase3 in KGN cells induced by DHEA were counteracted by *TOMM40*-OE treatment (Fig. 4C), suggesting that *TOMM40* had inhibitory effects on the apoptosis of ovarian granulosa cells stimulated by DHEA.

### *TOMM40* Plays a Significant role in the key Pathways Associated with PCOS and Immunity in Tumors Cancers

**Fig. 5:**
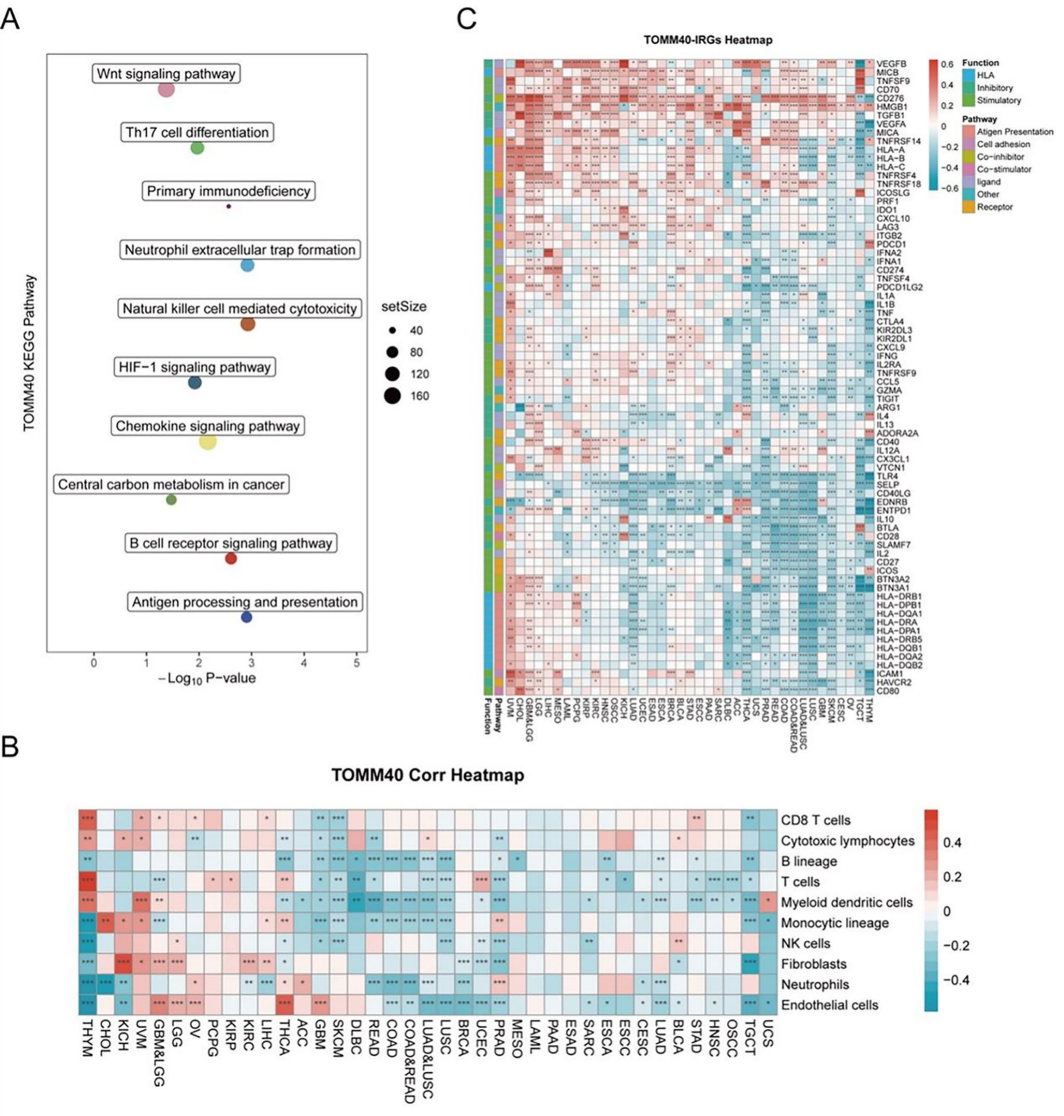
KEGG analyses perform functional enrichment analyses of *TOMM40* in PCOS. **(A)** The KEGG enrichment analysis identified the top 10 molecular functions of DEGs. **(B)** The simultaneous expression of *TOMM40* and genes related to the immune system can be observed. **(C)** In various types of cancer, the correlation between *TOMM40* expression and the infiltration of immune cells is depicted. *P < 0.05, ** *P* < 0.01 or *** *P* < 0.001

GSEA established the KEGG analysis of *TOMM40*. As illustrated in Fig. 5A. According to the findings, the *TOMM40*-related genes played a significant role in immune response and cancer signaling pathways, including Th17 cell differentiation, Natural killer cell mediated cytotoxicity, B cell receptor signaling pathway, Wnt signaling pathway, and Central carbon metabolism in cancer (*P* < 0.01). Based on CIBERSORT analysis, there was an association between immune cell infiltration and *TOMM40* expression in several types of cancer (Fig. 5B). In various types of cancers, the expression level of *TOMM40* showed a positive correlation with CD8 T cells, T cells and Endothelial cells. Additionally, a co-expression analysis was conducted on 33 tumors to identify the associations between *TOMM4*0 expression, and genes related to the immune system. Based on the heat map (Fig. 5C), it is evident that nearly all immune-related genes exhibited co-expression with *TOMM40*. Except for LUSC, LUAD, COAD, READ, PRAD, UCEC and SARC, most immune-related genes displayed a positive correlation with *TOMM40* across all tumor types (*P* < 0.05).

### Pan-Cancer *TOMM40* Expression

**Fig. 6:**
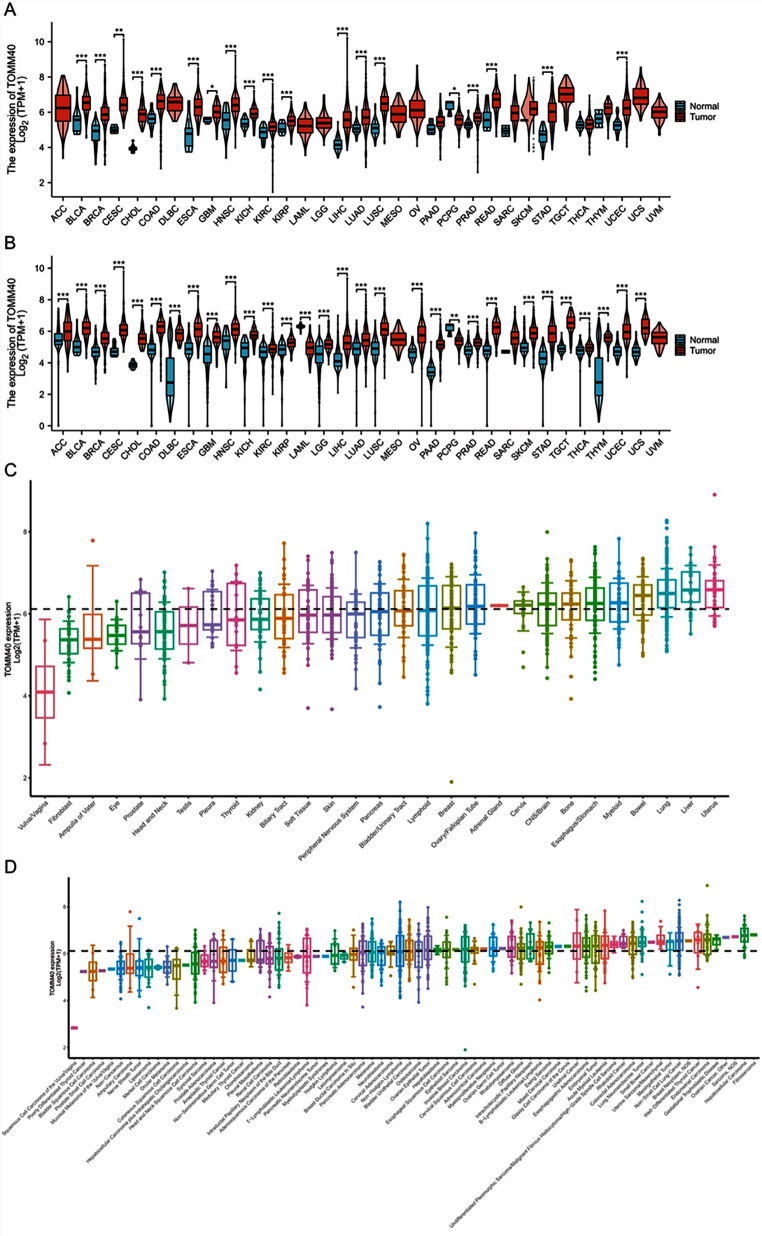
Pan-cancers exhibit *TOMM40* expression. **(A)** The TCGA dataset contains pan-cancer expression levels of *TOMM40*. **(B)** Expression levels of *TOMM40* in the TCGA and GTEx datasets across multiple cancers. **(C-D)** Expression of *TOMM40* in different cell lines and tissues. *P < 0.05, ** *P* < 0.01, *** *P* < 0.001. ^NS^*P*, no significance

**Fig. 7:**
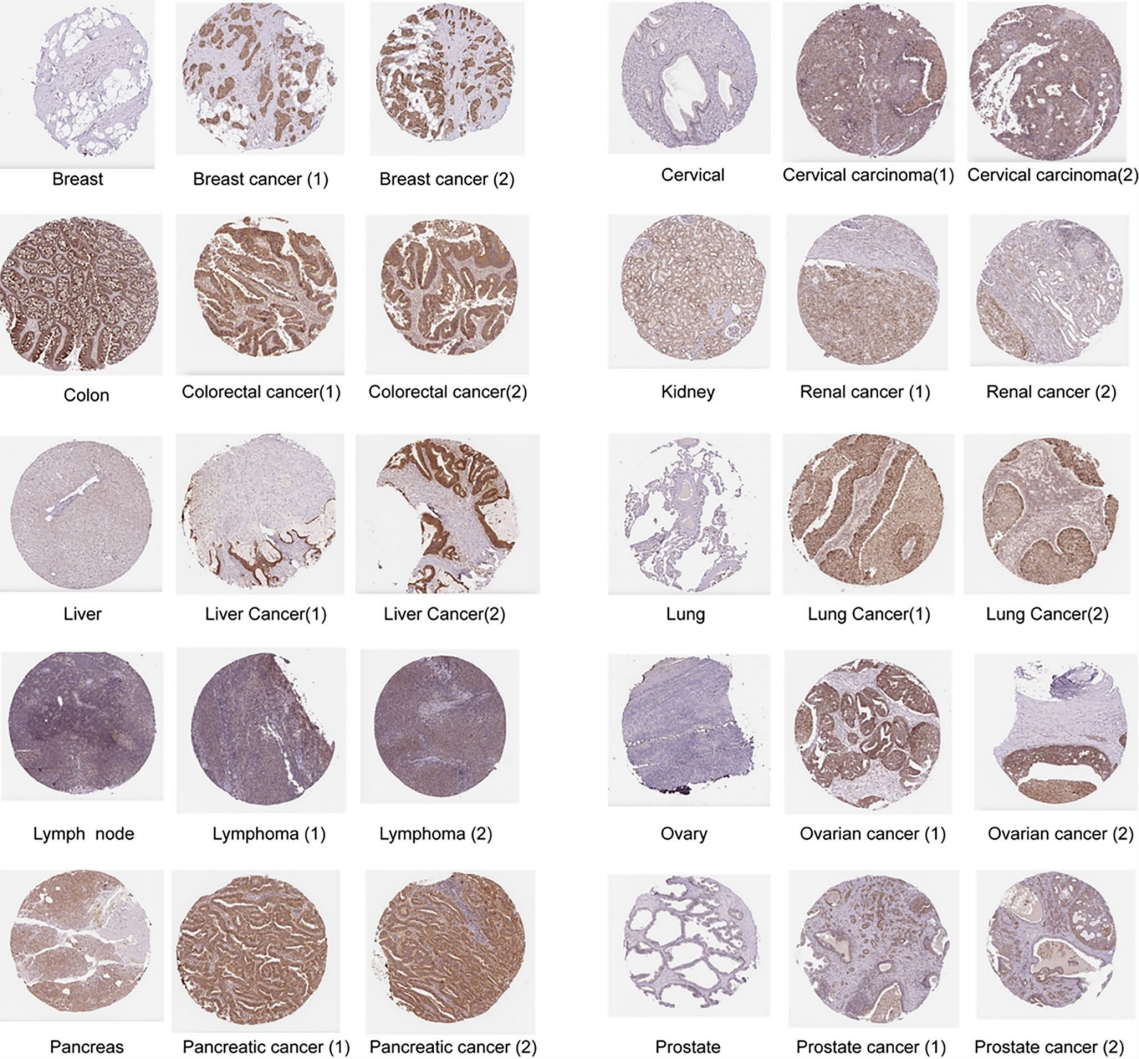
The protein expression of *TOMM40* in both the normal (on the left) and tumor (on the right) groups

In the TCGA data (Fig. 6A), *TOMM40* exhibited high expression in BRCA, BLCA, CESC, CHOL, COAD, ESCA, GBM, KICH, KIRC, KIRP, HNSC, LIHC, LUAD, LUSC, PRAD, READ, STAD and UCEC, while showing low expression in PCPG In addition, we obtained normal tissue data from the GTEx database and found that *TOMM40* exhibited low expression levels in ACC, BLCA, BRCA, CESC, CHOL, COAD, DLBC, ESCA, GBM, HNSC, KICH, KIRC, KIRP, LGG, LIHC, LUAD, OV, PAAD, PRAD, READ, SKCM, STAD, TGCT, THCA, THYM, UCEC, UCS, while showing low expression levels in LAML, and PCPG (Fig. 6B). According to the data, *TOMM40* was observed in both the cell lines and organ cells (Fig. 6C-D). Additionally, we utilized the HPA database to acquire immunohistochemical images to assess the protein level of *TOMM40*. Based on Fig. 7, it is evident that *TOMM40* protein exhibited significantly elevated expression in 10 types of cancer compared to its expression in normal tissues.

The analysis of the operating system showed that the correlation between *TOMM40* expression and OS was significant in four types of cancer, namely LGG, LIHC, LUAD, and SKCM, acting as protective factors (Fig. S3 A-I). The analysis conducted in DSS showed a significant association between *TOMM40* expression and DSS in 5 types of cancer (ACC, KIRP, LGG, LUAD, and SKCM), where *TOMM40* expression acted as a protective factor (Fig. S4 A-I). The PFS study found that the correlation between *TOMM40* expression and PFS was significant in 6 types of tumors, including ACC, LGG, LUAD, PRAD, and UVM (Fig. S5 A-I), indicating a protective effect.

### Evaluation of *TOMM40*’s Significance in Various Types of Cancer

**Fig. 8:**
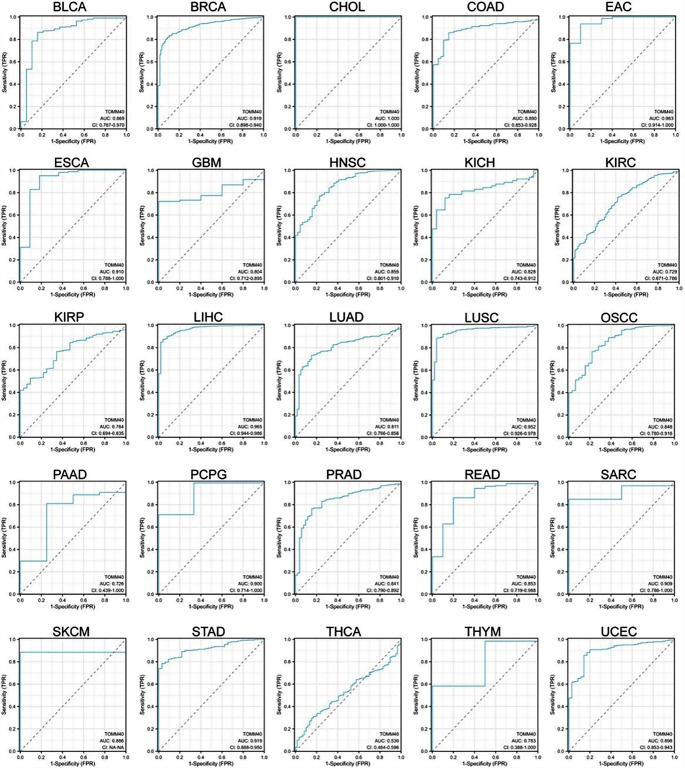
The diagnosis performance of *TOMM40* in the TCGA cohort was confirmed by the AUC of ROC curves

During the assessment of the tumor stage correlation, we observed a notable rise in *TOMM40* expression in the initial tumor stage (Fig. 8) across 16 cancer types, namely CHOL, LUSC, LUAD, KIRP, HNSC, LIHC, ESCA, KIRC, UCEC, BLCA, COAD, READ, STAD, PRAD, THCA, and BRCA. This suggests that *TOMM40* could hold significant clinical significance in the early detection of these malignancies. To assess the diagnostic accuracy of the gene signature, the performance was evaluated using ROC curves. Various AUC thresholds have been contemplated to signify excellent diagnostic precision (AUC 1.0–0.9), moderate diagnostic precision (AUC 0.9–0.7), or poor diagnostic precision (AUC 0.7–0.5). In Fig. 8, it can be observed that the model’s ROC analysis demonstrates high diagnostic accuracy in 9 cancer types, moderate diagnostic accuracy in 17 cancer types, and low diagnostic accuracy in 1 cancer type. It is important to highlight that the AUC attained a perfect score of 1.0 in CHOL.

## Discussion

PCOS, a condition affecting the female reproductive system, has negative effects on women’s endocrine system, reproductive health, and metabolism. Based on our understanding, individuals diagnosed with PCOS are at an elevated susceptibility to various types of cancer, including [5, 28], breast cancer [29], thyroid cancer [30], and other forms of cancer [31]. Expressed throughout the body, *TOMM40*, the largest member of the TOMM family, exhibits increased levels in the ovaries of females. In a previous study, *TOMM40* was found in bovine oocytes [32]. Furthermore, numerous research has indicated that *TOMM40* functions as a cancer-causing gene in malignancies and enhances the advancement of tumors [15, 33]. Hence, targeting the fundamental pathophysiological connection between PCOS and cancers could be crucial in developing a clinical treatment plan for either PCOS or cancers.

The present study employed two machine learning techniques, namely LASSO and SVM-RFE models, to identify three potential target genes (MAP1LC3A, *TOMM40*, and VDAC1) associated with PCOS. The average AUC values of MAP1LC3A, *TOMM40*, and VDAC1 were 0.809, 0.715, and 0.745, respectively, as indicated by our data analysis. Based on our current understanding, MAP1LC3A has been demonstrated to play a significant role in the progression of PCOS [34]. And also, Sahar Mazloomi et al. [35], report that VDAC1 was significantly lower in PCOS patients. The focus of our data lies in examining the role and impact of *TOMM40* in PCOS. Our findings indicate a significant decrease in *TOMM40* expression within granulosa cells from patients with PCOS, ovarian tissue obtained from a mouse model of PCOS, as well as DHEA-induced KGN cells within our dataset. The presented evidence suggests a significant contribution of *TOMM40* to the progression of PCOS, particularly in granulosa cells. Therefore, we conducted molecular biology experiments to investigate the functional role of *TOMM40* in KGN cells. Our findings demonstrate that exogenous overexpression of *TOMM40* in KGN cells effectively reverses the apoptosis rate induced by DHEA.

The two predominant categories of illnesses globally are PCOS and various types of cancers, which exhibit common features associated with hormonal and endocrine imbalances. Hence, it is plausible to hypothesize that PCOS biomarkers could potentially be utilized in strategies for tumor prediction. To screen the potential biological markers, we conducted two types of machine learning. The analysis of *TOMM40* in PCOS revealed that its metabolic pathways are associated with cancer-related pathways, such as the Wnt signaling pathway, HIF-1 signaling pathway, and central carbon metabolism in cancer. Additionally, it is also related to immune pathways including Th17 cell differentiation, Primary immunodeficiency, Nature killer cell mediated cytotoxicity, and B cell receptor signaling pathway. According to our current understanding, Wnt signaling pathway plays a crucial role in the development of cancers [36]. Furthermore, Bose S suggested that cancer progression is influenced by the cytotoxicity mediated by natural killer cells [37, 38]. These pieces of evidence indicate that *TOMM40* significantly contributes to both PCOS and cancer.

Given the strong correlation between PCOS, inflammation, immunity, and endothelial activation, we were motivated to utilize bioinformatics pan-cancer database analysis techniques to investigate the involvement of *TOMM40*, a gene associated with PCOS, in various types of cancer. Our analysis unveiled a significant oncogenic expression of *TOMM40* across various cancer types (Fig. 5). To comprehend the potential impact of PCOS on TMB (Tumor Mutation Burden), we investigated the presence of *TOMM40* in cancer infiltration. Our findings have revealed a robust positive correlation between *TOMM40* and the extent of immune infiltration by diverse immune cells, as well as TMB (Figs. 6 and 7). This further supports our subsequent analysis investigating the association between *TOMM40* expression and the clinical characteristics of the subjects. Afterwards, we investigated the correlation between *TOMM40* expression and the clinical characteristics of the patients. The aim of our investigation was to establish the correlation between *TOMM40* expression and the clinical characteristics of the patients (Fig. S3-5). By referencing our current study, we successfully established a significant association between *TOMM40* expression and the clinical features of the patients. Our analysis revealed a significant correlation between *TOMM40* expression and the clinical features of patients, as well as its impact on tumor-associated overall survival (OS), disease-specific survival (DSS) and progression-free survival (PFS). Notably, lower levels of *TOMM40* expression were associated with steeper downward trends in the survival curve and increased mortality rates (Fig. 8). According to the study findings, it is hypothesized that *TOMM40* associated with PCOS could potentially serve as a prognostic indicator for various types of cancer.

Based on the bioinformatics discoveries of PCOS and pan-cancer, our conclusion is that *TOMM40* could potentially function as a potential -biomarker for the prediction and diagnosis of both PCOS and pan-cancer. Researching *TOMM40* could lead to the discovery of novel markers and indicators for advanced clinical liquid biopsy, while also contributing to the prevention of PCOS and tumors to some degree.

### Limitation of the Study

The results of this study were obtained from public database and PCOS model, as well as vitro study. Having an in-depth understanding of the role of immunity and the development of cancer in human would advance our understanding of *TOMM40* as a factor in follicle development and PCOS.
